# Association between clinical and pathological factors and risk of radioiodine refractory in patients with differentiated thyroid carcinoma

**DOI:** 10.3389/fendo.2026.1709736

**Published:** 2026-03-11

**Authors:** Aidana Rakhmankulova, Laura Pak, Lyudmila Pivina, Zhandos Burkitbayev, Andrey Orekhov, Diana Pak, Saltanat Bolsynbekova, Maksim Pivin, Dana Seitkhanova, Kairzhan Kudaiberdinov, Altay Dyussupov, Masahiro Nakashima

**Affiliations:** 1 Semey Medical University, Semey, Kazakhstan; 2 National Research Oncology Center, Astana, Kazakhstan; 3 Astana Vision, Astana, Kazakhstan; 4 Medical Centre Hospital of the President’s Affairs Administration of the Republic of Kazakhstan, Astana, Kazakhstan; 5 Head of the Department of Oncology and Molecular Diagnostic Pathology, Nagasaki University, Nagasaki, Japan

**Keywords:** case-control study, differentiated thyroid carcinoma, radioiodine resistance, radioiodine therapy, risk factors

## Abstract

**Background:**

Currently, the therapeutic treatment of differentiated thyroid carcinoma (DTC) is based on the use of radioactive iodine; the effectiveness of treatment depends on the sensitivity of tumor cells to therapy. Factors associated with a high risk of radioactive iodine resistance of DTC (RAIR-DTC) are poorly understood in the current literature, but understanding their role may help optimize patient care. The aim of our study is to assess the relationship between the clinical and pathological characteristics of DTC and the risk of radioiodine resistance.

**Methods:**

We conducted a case-control study involving a targeted sample of patients with differentiated thyroid carcinoma (DTC). The study included a total of 373 patients, of whom 60 were radioiodine-resistant and 313 were radioiodine-sensitive. For the molecular analysis, an additional sub-cohort was selected from the overall sample (n = 167), in which mutations in BRAF V600E, NRAS (codon 61) and pTERT (C228T/C250T) were determined using ddPCR.

**Results:**

In the group of patients with RAIR-DTC, total thyroidectomy with radical lymph node dissection was performed twice as often, which indicates more aggressive tumor invasion in this category of patients (p<0.001). The main risk factors for RAIR-DTC were female gender, total thyroidectomy with radical lymph node dissection, the presence of metastases in the lymph nodes, the total radiation dose, the absence of distant metastases, and the total number of lymph nodes removed, in the histological subcohort (n = 167), the presence of the double BRAF+pTERT mutation was also identified. Multivariate regression analysis showed that statistically significant risk factors for radioiodine resistance were the total radiation dose, the absence of distant metastases, and the total number of removed lymph nodes. These results were confirmed by ROC analysis; AUC was 0.796 (95% CI 0.726-0.865), p<0.0001.

**Conclusions:**

The obtained data highlight the interplay between clinical and molecular factors in the development of radioiodine resistance in differentiated thyroid cancer (DTC). The co-occurrence of BRAF and TERT mutations has potential prognostic significance. These findings suggest that integrating clinical and molecular data enables more accurate risk stratification for radioiodine resistance and helps define the direction of future research.

## 1 Introduction

The incidence and prevalence of thyroid cancer has increased significantly worldwide in recent decades. This is attributed to the intensification of the impact of adverse environmental factors, including radiation exposure and industrial risk factors, as well as the role of genetic predisposition (1, 2). Furthermore, there has been an increase in mortality from thyroid cancer, with an average annual increase of approximately 1.1%, and even higher rates of increase of 2.9% per year at advanced stages of papillary thyroid cancer (3). Between 1990 and 2019, The global mortality rate from thyroid cancer increased from 2.01 to 2.83 per 100,000 people (4).

It is known that more than 90% of all malignant thyroid neoplasms are differentiated thyroid carcinomas (DTC), which include the papillary type (about 84% of all carcinoma cases) and the follicular type, according to histopathological characteristics (5, 6). The most common cause of severe disease progression and death from thyroid carcinoma is the recurrent course of the disease with the development of distant metastases, which occur in 4–23% of cases, with lungs being the primary site for localization (7, 8).

The main methods of treating DTC remain surgical treatment and radioactive iodine therapy; moreover, in cases where tumor progression with the spread process in the form of metastasis is observed, radioiodine therapy becomes the first-line treatment (9, 10). The steady increase in the number of patients with DTC and subject to radioiodine therapy requires an analysis of the complications and long-term consequences of this treatment.

At present, the therapeutic treatment of thyroid carcinoma is based on the use of radioactive iodine, and the effectiveness of treatment depends on the sensitivity of tumor cells to this agent. However, evaluation of the effectiveness of patient treatment shows that in a large number of cases, tumors are unable to retain iodine, that is, they become resistant to treatment, which leads to unfavorable outcomes (11). It is known that despite the appointment of adequate treatment to patients, about 20-40% of patients with DTC develop recurrences during treatment and observation, particularly in the presence of metastases (12, 13). Only about 30% of patients with distant metastases have a chance to achieve stable remission through radioactive iodine therapy (14). The remaining patients are at high risk of developing resistance to such therapy at the beginning of treatment or after several courses of radioiodine therapy (14). The incidence of refractoriness to radioactive iodine therapy ranges from 2 to 5% of all DTC cases (15); in this case, first-line therapeutic agents are tyrosine kinase inhibitors (16).

One of the main mechanisms of resistance to radioactive iodine in differentiated thyroid carcinoma is the absence of the sodium iodide symporter in the basement membrane of follicular cells, which is necessary for iodine uptake. This may be associated with gene mutation or abnormal activation of signaling pathways, which leads to impaired gene expression and cell resistance to radioactive iodine therapy (17). The sodium iodide symporter (NIS) is a plasma membrane glycoprotein located on the basolateral surfaces of thyroid follicular epithelial cells, which mediates active iodide transport into thyroid follicular cells. The loss of NIS is largely associated with molecular abnormalities, including activating BRAF mutations (primarily BRAF V600E) and subsequent activation of the MAPK signaling pathway, leading to decreased expression of SLC5A5 (NIS) and thyroid-specific transcription factors (PAX8, TTF-1). Additionally, abnormal activation of PI3K/AKT- and TGF-β-dependent pathways, as well as epigenetic changes such as hypermethylation of the SLC5A5 promoter, exacerbate NIS expression disruption and contribute to the formation of a radioiodine-resistance tumor phenotype. A correlation has been established between the loss of cell differentiation and the development of radioiodine resistance with the degree of activation of the mitogen-activated protein kinase pathway, which increases in tumors with BRAF (B-Raf proto-oncogene) mutations (11).

Factors associated with a high risk of radioiodine resistance in thyroid carcinoma (RAIR) DTC have not been sufficiently studied in the modern literature; however, understanding the role of these factors may not only help in assessing the mechanisms and chances of developing this resistance but also contribute to optimization of patient treatment.

The aim of the study is to assess the association between the clinical and pathological characteristics of differentiated thyroid carcinoma and the risk of developing radioiodine resistance.

## 2 Materials and methods

### 2.1 Sample description

The case-control study was conducted among patients who received treatment in the Radionuclide Therapy Department of the Semey Center for Nuclear Medicine and Oncology (Republic of Kazakhstan). This department is the only one in the Republic of Kazakhstan that provides radioiodine therapy for thyroid carcinoma. Primary information was collected on the basis of the unified electronic database of the medical information system, which includes oncology patients. For the analysis, a database of patients who underwent radioiodine therapy was formed, including 630 patients who underwent treatment for highly differentiated thyroid carcinoma in the period from January 2021 to December 2023. Of these, 373 medical records met the established inclusion criteria. The database was cleared of cases where the necessary laboratory and diagnostic data for analysis were missing.

During the entire study period, radioiodine therapy for thyroid carcinoma was administered to 373 patients. The group of patients with radioiodine-resistant differentiated thyroid cancer (RAIR-DTC) comprised 60 patients, each of whom met at least one criterion of radioiodine refractoriness. These criteria were not mutually exclusive, and one patient could meet more than one of them. Specifically, 9 patients (15%) had at least one focus that did not accumulate therapeutic activity of radioactive iodine; 23 patients (38.3%) had structural progression of the tumor process within 12 months of radioiodine therapy; and 27 patients (45%) had no tumor regression with a total therapeutic activity of more than 22 GBq (600 mCi). The control group comprised 313 patients who responded adequately to radioiodine therapy (radioiodine sensitivity).

**Figure 1:**
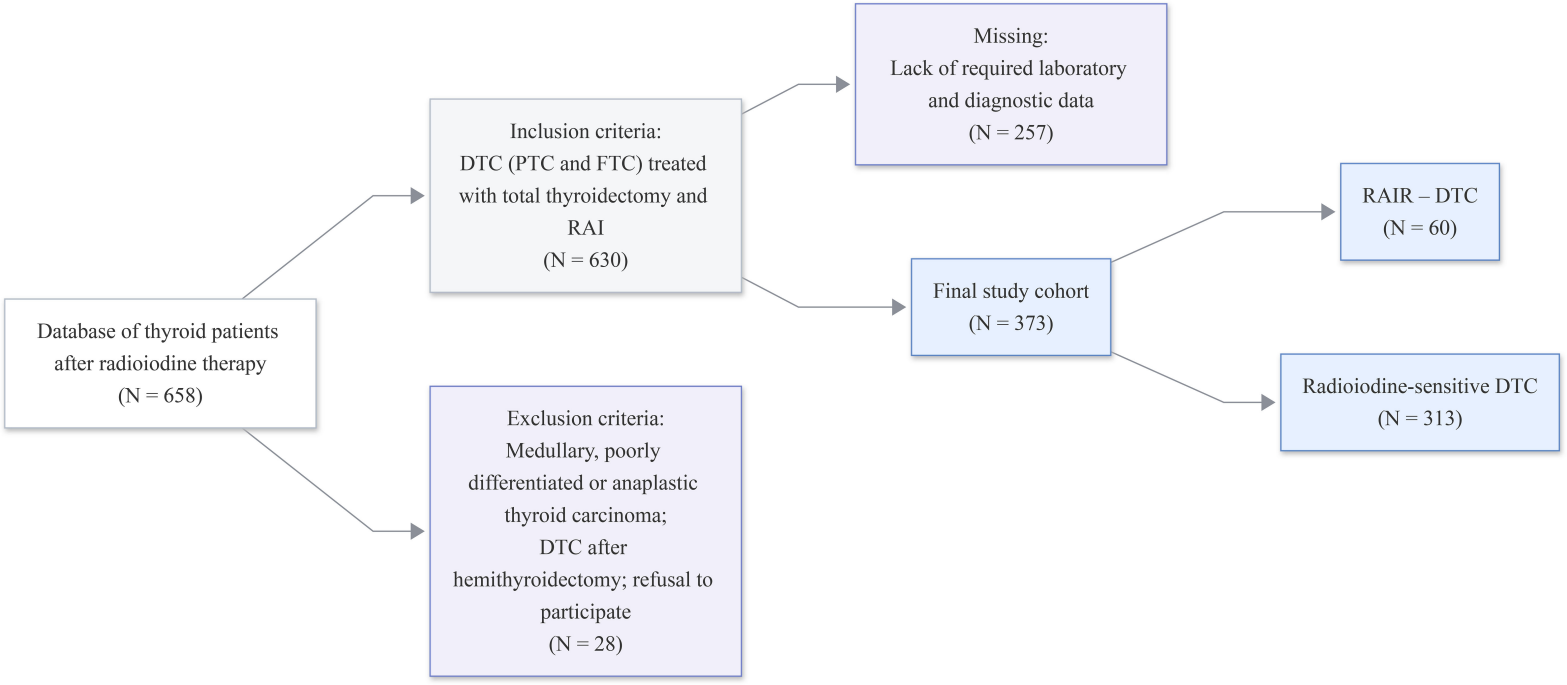
Flow chart of the study.

The scheme of patient selection for the study is presented in **Figure 1**.

**Figure 2:**
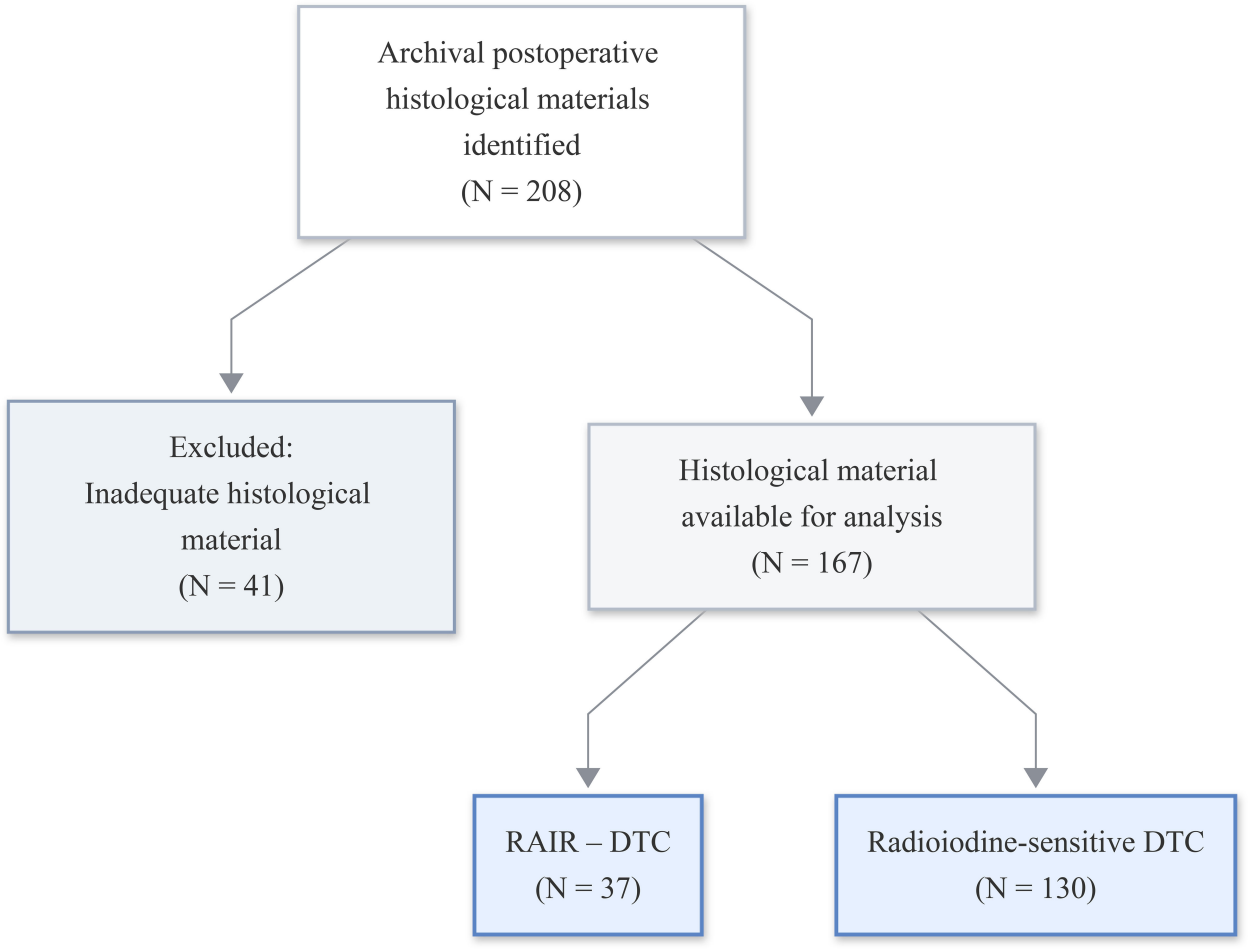
Flow chart of the histological subcohort.

A subcohort of patients was formed from the final clinical cohort for morphological and molecular analyses, where archival histological materials (histological slides and paraffin blocks) were available. Histological slides and paraffin blocks were identified for 208 patients. However, in 41 cases, the material was deemed unsuitable for analysis due to insufficient tissue volume, sectioning artefacts or suboptimal fixation. The final analytical subcohort comprised 167 patients, who were categorized as either radioiodine-refractory differentiated thyroid cancer (RAIR-DTC, n = 37) and radioiodine-sensitive differentiated thyroid cancer (n = 130). **Figure 2** illustrates the subcohort selection process.

Inclusion Criteria refers to patients of both genders diagnosed with well-differentiated thyroid carcinoma (papillary or follicular forms) who underwent surgery of total thyroidectomy or total thyroidectomy with compartment-oriented fascial excision of the neck tissue, aged from 18 to 80 years; undergoing radioiodine therapy during the specified period; patient data, the volume of which was sufficient and necessary for conducting our study.

Exclusion Criteria refers to patients with medullary, poorly differentiated or anaplastic thyroid carcinoma, patients with differentiated thyroid carcinoma who underwent hemithyroidectomy; refusal to participate in the study;

Missing patients: patients whose clinical data did not meet the minimum requirements necessary for this study.

### 2.2 Ethical aspects

This study was approved by the Local Ethics Committee of the Semey Medical University NCJSC (extract from the Minute No. 1b dated November 2, 2023). In addition, before admission to the hospital, patients signed Patient Informed Consent for the anonymized distribution of their medical information. The confidentiality of each participant was maintained by adhering to an ethical code of conduct.

### 2.3 Methodology for conducting radioiodine therapy

In differentiated thyroid carcinoma, a mandatory condition for performing radioiodine therapy is complete surgical removal of the thyroid gland (total thyroidectomy) and an increase in the level of thyroid-stimulating hormone (TSH) in the blood to values exceeding 30 mU/l (18). In the treatment of differentiated thyroid carcinoma, the calculation of I - 131 activity depends on the required therapeutic effect and is as follows: for ablation of residual tissue after surgical removal of the thyroid gland without metastases and infiltration into surrounding tissues - 3700 MBq (100 mCi); in the presence of metastases in regional lymph nodes and/or infiltration into surrounding tissues - 5550 MBq (150 mCi); in the presence of distant metastases to the lungs and bones - from 7400 to 11100 MBq (200–300 mCi) (19).

### 2.4 Criteria for resistance to radioiodine therapy

Radioiodine resistance was determined based on post-therapy whole-body scintigraphy and whole-body positron emission tomography (PET-CT) data according to the following criteria for radioiodine resistance (20):

- The presence of one or more foci of well-differentiated thyroid carcinoma that are visualized on positron emission tomography but show no I-131 uptake on post-therapeutic whole-body scintigraphy;
- Progression of the tumor process within ≤ 12 months during radioiodine therapy with activities of at least 3.7 GBq, provided that the thyroid remnant ablation is successfully achieved;
- Absence of regression of tumor foci with a cumulative therapeutic activity of radioactive iodine exceeding 22 GBq (600 mCi).

Predictors of radioiodine refractoriness also included an increase in the level of thyroglobulin and antibodies to thyroglobulin without evident structural progression; significant uptake of 18F-FDG in PET-CT by metastatic foci; and the absence of accumulation of I-131 according to PET/CT data upon administration of therapeutic or diagnostic activity.

Moreover, according to the 2015 American Thyroid Association (ATA) guidelines, radioiodine-refractory differentiated thyroid carcinoma (RAI-R DTC) is divided into four categories (17, 19): primary RAI-R DTC, characterized by the initial inability of the tumor to absorb radioiodine; secondary RAI-R DTC, in which the loss of the ability to accumulate radioiodine occurs at later stages of the disease; heterogeneous RAI-R DTC, characterized by heterogeneity of radioiodine uptake in different tumor sites, with some lesions retaining the ability to accumulate it, while others lose it; progressive RAI-R DTC, in which the disease continues to progress despite the preserved ability of the tumor to absorb radioiodine.

### 2.5 Treatment procedure and follow-up

A prerequisite for radioiodine therapy is a mandatory prior total thyroidectomy. In this case, the TSH level in the blood should be ≥30 mU/l, for which levothyroxine is discontinued 4 weeks before the start of radioactive iodine therapy. On the first day of hospitalization, the patient receives I-131 orally in a dose in accordance with the individual patient’s status. On the fourth day of hospitalization, the patient undergoes post-therapeutic whole-body scintigraphy. The results of the scintigraphy are discussed collegially by specialists within the multidisciplinary group, leading to a conclusion. Patients are given recommendations for subsequent whole-body scintigraphy after 6 months to assess treatment efficacy or PET/CT with 18-FDG for differential diagnosis and to exclude radioiodine-refractory foci. A repeated course of radioactive iodine treatment is prescribed 6 months after the initial treatment if serum thyroglobulin levels remain elevated or in accordance with the results of post-therapeutic whole-body scintigraphy. In case of recurrence, tumor invasion, or the presence of distant metastases, an oncologic surgeon is consulted to determine the feasibility of surgical intervention.

### 2.6 Surgical tactics and completeness of resection

All patients included in the study underwent total thyroidectomy or total thyroidectomy with compartment-oriented fascial excision of the neck tissue. Thus, complete surgical resection was achieved in all cases. Operative and pathological reports were analyzed to confirm the extent of thyroidectomy and the absence of residual thyroid tissue.

### 2.7 Determination of thyroglobulin levels

Serum thyroglobulin (Tg) levels (ng/mL) were measured using an immunoassay method after total thyroidectomy and radioiodine therapy. Thyroglobulin was considered a biochemical marker of preserved or persistent thyroid tissue in the absence of radioiodine uptake on whole-body scintigraphy. In accordance with clinical practice adopted in the Republic of Kazakhstan, Tg levels were evaluated dynamically during follow-up, either under thyroid-stimulating hormone (TSH) suppression or after levothyroxine withdrawal, depending on the clinical situation. A persistently elevated thyroglobulin level over time, in the absence of radioiodine uptake, was interpreted as an indicator of radioiodine-refractory disease progression. Thyroglobulin is used to assess patients for disease progression and to inform decisions regarding the type and timing of further interventions considered appropriate (21).

### 2.8 Pathomorphology and TNM staging

All histological specimens were evaluated by two pathologists. Subtypes and variants of differentiated thyroid carcinoma were classified according to the WHO Classification of Endocrine Tumors (5th Edition, 2022) (22). TNM staging was performed in accordance with the AJCC 8th edition. Capsular and vascular invasion were recorded quantitatively. Positive surgical margins were defined by the presence of tumor cells in the ink-marked area. Lymph nodes were evaluated by anatomical levels, indicating the number examined and the number with metastatic involvement, as well as the presence of extracapsular extension.

Tumor staging was performed according to the TNM system following international standards outlined in the AJCC/UICC 8th edition (2017). For consistency, all cases were reclassified according to this version, with pT, pN, pM, and overall stage group determined for each patient. Operative definitions, including criteria for lymph node metastasis and micro or gross extrathyroidal extension, corresponded to AJCC-8 standards and were harmonized with the NCCN Clinical Practice Guidelines in Oncology: Thyroid Carcinomas, Version 2.2022 (23).

### 2.9 Molecular genetic analysis

Molecular analysis was performed on a histological subcohort of patients for whom postoperative archival material was available (n = 167). Mutation detection was carried out using droplet digital polymerase chain reaction (ddPCR; Droplet Digital PCR, USA) in a laboratory at the Atomic Bomb Disease Institute, Nagasaki University (Nagasaki, Japan). The analysis included the BRAF V600E mutation, the NRAS mutation at codon 61, and the TERT promoter mutations C228T and C250T.

### 2.10 Evaluation of results

All the data that could potentially act as risk factors for developing resistance to radioiodine therapy were analyzed. These factors included sex, age, body mass index, occupational exposure, the presence of chronic thyroid disease and other comorbidities, the postoperative histological diagnosis, the presence of invasion, disease stage, the presence of distant metastases, the cumulative administered activity of radioiodine therapy, post-therapeutic PET/CT findings, the time to disease recurrence, the BRAF V600E mutation, the NRAS mutation (codon 61) and the TERT promoter mutations (C228T and C250T). Prognostic outcomes were obtained from follow-up data available in the patient database.

### 2.11 Statistical analysis

Statistical data processing was performed using SPSS Statistics 20.0. Quantitative variables were assessed for normality using the Kolmogorov–Smirnov test. Quantitative variables with normal distribution were described using mean values (M) and standard deviations (SD), and 95% confidence intervals (95% CI); comparisons were performed using the Student’s t-test for independent samples. In the absence of normal distribution, quantitative data were described using the median (Me) and the lower and upper quartiles (Q1 - Q3). Comparison of two groups for quantitative variables with non-normal distribution was performed using the Mann–Whitney U test. Categorical data were described as absolute numbers and percentages, and associations between nominal variables were assessed using Pearson’s χ² test. Univariate and multivariate regression analysis were performed to identify factors influencing the risk of developing RAIR. The discriminative ability of the multivariate model was evaluated using ROC analysis, with the construction of the ROC curve and calculation of the area under the curve (AUC). The optimal cutoff value was determined using Youden’s index (sensitivity + specificity − 1). For the selected cutoff value, sensitivity, specificity, positive predictive value (PPV), and negative predictive value (NPV), as well as the corresponding confidence intervals, were calculated.

## 3 Results

### 3.1 Population characteristics

The social and demographic characteristics of the study groups, depending on their sensitivity to radioactive iodine therapy, are presented in **Table 1**. The study groups were age-compatible (the average age of the study subjects was 50 (38-62) years, of which 174 patients (46.5%) were aged over 50). The minimum age of the study subjects at the time of inclusion in the study was 20, the maximum was 86, while the minimum age at the time of debut was 15, the maximum age was 76. Women predominated in the structure of patients in both the main and control groups, but it should be noted that in the group of people resistant to radioiodine therapy, the number of men was twice as high as in the control group (p = 0.011). About a quarter of patients in both study groups had a burdened history of thyroid diseases before the development of thyroid carcinoma. The body mass index on average in both study groups was in the overweight range. None of the patients exceeded the threshold for grade I obesity. Regarding occupational conditions, 12 people (3.2%) worked in hazardous industries (associated with the influence of industrial risk factors), and five of them (41.6%) were radioiodine-refractory. A total of 83 patients (22.3%) were retired, 76 patients (20.4%) were unemployed, 16 people (4.3%) were disabled, and 185 (49.7%) patients were employed.

**Table 1:** The social and demographic characteristics of the study groups, depending on their sensitivity to radioactive iodine therapy.

Parameter	Total number of patients, n= 373	Study groups		p
		RAI-sensitivity DTC, n= 313	RAIR-DTC n= 60	
Age (median), years	50.0 (38-62)	50.0 (39.0-62.0)	47.5 (36.75-62.25)	0.679
Age, n (%):				
< 50 years > 50 years	199 (53.4) 174 (46.6)	167 (53.4) 146 (46.6)	32 (53.3) 28 (46.7)	0.998
Gender, n (%):				
women men	316 (84.7) 57 (15.3)	272 (86.9) 41 (13.1)	44 (73.2) 16 (26.8)	0.011
History of TC, n (%)	89 (23.9)	72 (23.0)	17 (28.3)	0.375
BMI, kg/m2 (median)	27.1 (23.9-31.5)	27.01 (23.9-31.5)	27.55 (24.15-31.52)	0.791
Age of TC debut (median), years	45.0 (34.0-57.0)	45.0 (34.0-57.0)	43.0 (31.75-57.0)	0.305

Resume: The groups were comparable in terms of age and body mass index, with a predominance of women. However, the proportion of men was significantly higher among patients with radioiodine resistance, and approximately a quarter of patients had a history of thyroid disease.

### 3.2 Clinical and pathological characteristics of patients with malignant neoplasms of the thyroid gland

In the main group of patients, the thyroglobulin level was significantly higher compared to the control group (23.95 ng/mL vs 2.00 ng/mL, p < 0.001). Anti-Tg antibody titers were also higher in the main study group (16.41 IU/mL vs 13.2 IU/mL, p = 0.011). Elevated levels of thyroglobulin and anti-thyroglobulin antibodies in radioiodine-refractory patients may indicate the presence of a considerable volume of tumor tissue that is unable to accumulate radioiodine, which reflects a biologically more aggressive course of the disease and reduced effectiveness of radioiodine therapy. When assessing the extent of surgery performed for thyroid carcinoma, it was found that total thyroidectomy with radical lymph node dissection was performed for 58.3% of patients in the main group, characterized by radioiodine refractoriness to RIT, whereas in the control group, this extent of surgery was applied in only 29.1% of cases (p < 0.001). The predominant histological types of thyroid carcinoma in the study groups were papillary carcinoma (74.5%) and follicular carcinoma (25.5%). When analyzing the pattern of tumor invasion, no statistically significant differences were found between the radioiodine-sensitive and radioiodine-refractory groups (p = 0.072). The absence of invasion was observed in 26.8% of patients in the control group and 30.0% in the main group. Invasion into a single organ was detected in 41.2% of the control group and 31.7% of the main group, while multiple organ invasion was recorded in 31.9% and 38.3% of patients, respectively. A detailed analysis of the localization of multiple invasion showed that the greatest contribution to its structure was made by combined damage to the capsule and soft tissues — in 42 (11.3%) patients (10.9% in the RAI-sensitive group and 13.3% in the RAIR-DTC group). Less frequently, invasion of the capsule and vessels/nerves was noted in 27 (7.2%) patients (7.7% and 5.0%, respectively), as well as invasion of the capsule in 14 (3.8%) patients (3.2% and 6.7%, respectively). Combinations involving three or more structures (capsule, muscles, vessels/nerves, and soft tissues) were significantly less common, in most cases not exceeding 0.8–1.7% in both the overall cohort and in individual subgroups. Taken together, the data obtained indicate that in cases of multiple invasion, the tumor predominantly spread beyond the capsule, involving mainly soft tissues, vascular-nerve bundles. Thus, although multiple invasion was more frequent among radioiodine-refractory patients, the difference did not reach statistical significance. The presence of microcarcinoma in the context of these histological types was identified in 8 patients in the control group. The presence of metastases, including regional lymph node metastases, was significantly more frequent in the main group (23.3% vs. 7.3%) compared with the control group (p < 0.001) (**Table 2**).

**Table 2:** Clinical and pathological characteristics of patients with malignant neoplasms of the thyroid gland.

Parameter	Total number of patients, n= 373	Study groups		p
		RAI-sensitivity DTC, n= 313	RAIR-DTC n= 60	
Thyroglobulin (ng/mL)	2,09 (0,24-15,0)	2 (0,2-8,96)	23,95 (1,11-137,78)	< 0,001
Anti-Tg antibodies (IU/mL)	13,54 (1,82-22,44)	13,2 (1,51-20,5)	16,41 (3,98-51,89)	0,011
Operation extent, n (%):				
Total thyroidectomy	247 (66.2)	222 (70.9)	25 (41.7)	<0.00
Total thyroidectomy with radical lymph node dissection	126 (33.8)	91 (29.1)	35 (58.3)	<0.001
Histological type, n (%):				
Papillary carcinoma	278 (74.5)	234 (74.8)	44 (73.3)	0.816
Follicular carcinoma	95 (25.5)	79 (25.2)	16 (26.7)	0,072
Tumor invasion:				
No invasion	102 (27,3%)	84 (26,8%)		
Invasion into a single organ	148 (39,7%)	129 (41,2%)	18 (30,0%)	
Multiple organ invasion	123 (33,0%)	100 (31,9%)	19 (31,7%)	
Presence of microcarcinoma, n (%)	8 (2.1)	8 (2.6)	0	
Presence of metastases, including metastases to regional lymph nodes, n (%)	37 (9.9%)	23 (7.3)	14 (23.3%)	0.001
CTD (MBq/mCu)	5540.0 (3700.0 - 7400.0)/ 149.7 (100.0 – 200.0)	4440.0 (3700.0-6475.0)/ 120,0 (100.0 – 175.0)	8445.0 (5550.0-13592.0)/ 228.2 (150.0 – 367.4)	<0.001

CTD, cumulative total dose of radioiodine therapy .

Resume: The data obtained indicate that radioiodine-resistant patients are characterized by a higher tumor burden (in terms of TG and anti-TG levels) and more frequent extended surgical interventions, which reflects a biologically more aggressive course of the disease and can be considered as a combination of unfavorable prognostic signs.

**Table 3** presents the distribution of patients with differentiated thyroid carcinoma according to the TNM classification and disease stages. All patients underwent total thyroidectomy with or without compartment-oriented fascial excision of the neck tissue prior to radioiodine therapy. The most common tumor categories were T2 (38.6%) and T1 (27.9%), indicating predominantly intracapsular growth with limited extrathyroidal extension. Metastatic involvement of regional lymph nodes (N1) was detected in 35.4% of patients, while distant metastases (M1) were observed in 2.9% of cases. According to the AJCC/UICC classification, the majority of patients were classified as stage I (65.4%), less frequently as stage II (20.9%), stage III (9.1%), and stage IV (4.6%).

**Table 3:** Distribution of patients with differentiated thyroid carcinoma according to TNM categories and disease stages.

Parameter	All patients, n = 373		Study groups		р
	n	%	RAI-sensitivity DTC, n=313	RAIR-DTC n=60	
T- category					
T0	1	0,3	1 (0,3%)	0 (0%)	0,751
T1	105	28,2%	88 (28,1%)	17 (28,3%)	
T1a	1	0,3	1 (0,3%)	0 (0%)	
T1b	3	0,8	3 (1%)	0 (0%)	
T1c	1	0,3	1 (0,3%)	0 (0%)	
T2	144	38,6	120 (38,3%)	24 (40%)	
T3	95	25,5	82 (26,2%)	13 (21,7%)	
T3a	1	0,3	1 (0,3%)	0 (0%)	
T3b	4	1,1	2 (0,6%)	2 (0,6%)	
T4a	2	0,5	1 (0,3%)	1 (1,7%)	
Tx	2	0,5	2 (0,6%)	0 (0%)	
N-category					
N0	194	52	173 (55,3%)	21 (35%)	0,014
N1	130	34,9	103 (32,9%)	27 (45%)	
N1a	6	1,6	6 (1,9%)	0 (0%)	
N1b	4	1,1	2 (0,6%)	2 (3,3%)	
N2	2	0,5	1 (0,3%)	1 (1,7%)	
Nx	37	9,9	28 (8,9%)	9 (15%)	
M-category					
M0	340	91,2	286 (91,4%)	54 (90,0%)	0,569
M1	11	2,9	8 (2,6%)	3 (5%)	
Mx	22	5,9	19 (6,1%)	3 (5%)	
Disease stage					
Stage I	244	65,4	202 (64,5%)	42 (70,0%)	0,013
Stage II	78	20,9	73 (23,3%)	5 (8,3%)	
Stage III	34	9,1	27 (8,6%)	7 (11,7%)	
Stage IV	17	4,6	11 (3,5%)	6 (10%)	

Resume: The data obtained indicate that radioiodine-resistant patients are characterized by a higher tumor burden (in terms of TG and anti-TG levels) and more frequent extended surgical interventions, which reflects a biologically more aggressive course of the disease and can be considered as a combination of unfavorable prognostic signs.

### 3.3 Structure of the initial thyroid pathology in patients prior to the development of the oncological process

According to the data presented in **Table 4**, the most common thyroid pathology preceding the development of thyroid carcinoma was nodular or multinodular goiter, which was observed in more than half of the cases. In one-third of patients, thyroid carcinoma developed in the absence of any underlying thyroid disease. Chronic autoimmune thyroiditis and diffuse toxic goiter preceded the development of the oncological process quite rarely, constituting 1.6% and 2.7% of cases. No statistically significant differences were found between the study groups for any of the thyroid structure parameters.

**Table 4:** Structure of the initial pathology of the thyroid gland before the development of the oncological process.

Pathology	Total N (%)	RAI- sensitivity DTC, n=313 (%)	RAIR-DTC n=60 (%)	p
No	114 (30.6)	98 (31.3)	16 (26.7)	0.474 ^a^
Nodular goiter	204 (54.7)	172 (55.0)	32 (53.3)	0.818 ^a^
Multinodular goiter	12 (3.2)	11 (3.5)	1 (1.7)	0.457 ^in^
Diffuse toxic goiter	10 (2.7)	8 (2.6)	2 (3.3)	0.733 ^in^
Chronic autoimmune thyroiditis	6 (1.6)	4 (1.3)	2 (3.3)	0.246 ^in^
Endemic goiter	2 (0.5)	2 (0.6)	–	
Other	37 (9.9)	29 (9.3)	8 (13.3)	0.334 ^a^

a – X ^2^, ^b^– continuity correction (Yates’ criterion).

Resume: In most patients, thyroid cancer developed against a background of nodular and/or multinodular goiter, whereas chronic autoimmune thyroiditis and diffuse toxic goiter were rare; however, no statistically significant differences in the structure of background pathology were found between the study groups.

### 3.4 Analysis of risk factors for the development of radioiodine resistance and evaluation of the prognostic model

The logistic regression analysis performed allowed us to estimate the odds ratios for the development of radioiodine resistance in patients with DTC. In a univariate regression analysis, the risk of developing radioiodine resistance in men’ group was 2.412 times lower than in women, the differences in the odds ratio were statistically significant (OR = 0.415; 95% CI: 0.214 - 0.802), but in a multivariate regression analysis, this relationship with gender was statistically insignificant (p = 0.302). Total thyroidectomy with radical lymph node dissection was associated with a 1.506-fold increase in the risk of developing radioiodine resistance in univariate regression analysis (p<0.0001), while this association was not established in multivariate analysis (p=0.114). The presence of distant metastases in the lymph nodes was associated with the risk of developing radioiodine resistance in both univariate and multivariate regression analysis (p< 0.0001; p=0.021, respectively). The same situation was observed for the total dose, meaning that with an increase in dose, an increase in the risk of developing radioiodine resistance was observed (p< 0.0001; p<0.0001). The total number of removed lymph nodes showed a positive association with radioiodine refractoriness only in univariate regression analysis (p = 0.002) (**Table 5**).

**Table 5:** Logistic regression analysis of the risk of developing radioiodine resistance to RIT.

Parameter	Univariate analysis, OR (95% CI)	P	Multivariate analysis, OR (95% CI)	p
Male	0.415 (0.214 - 0.802)	0,009	0.657 (0.295-1.460)	0.302
Complete thyroidectomy with radical lymph node dissection	1.506 (1.246-1.82)​​​	< 0.0001	0.580 (0.295-1.139)	0.114
Total number of lymph nodes with metastases	1.16 (1.086-1.238)	< 0.0001	1.157 (1.022-1,.09)	0.021
Total number of removed lymph nodes	1.044 (1.016-1.074)	0.002	0.976 (0.915-1.040)	0.454
CTD GBK	1.231 (1.152-1.315)	< 0.0001	1.171 (1.088-1.260)	< 0.0001
Absence of distant metastases	0.261 (0.125-0.543)	< 0.0001	0.353 (0.117-1.0064)	0.054

**Figure 3:**
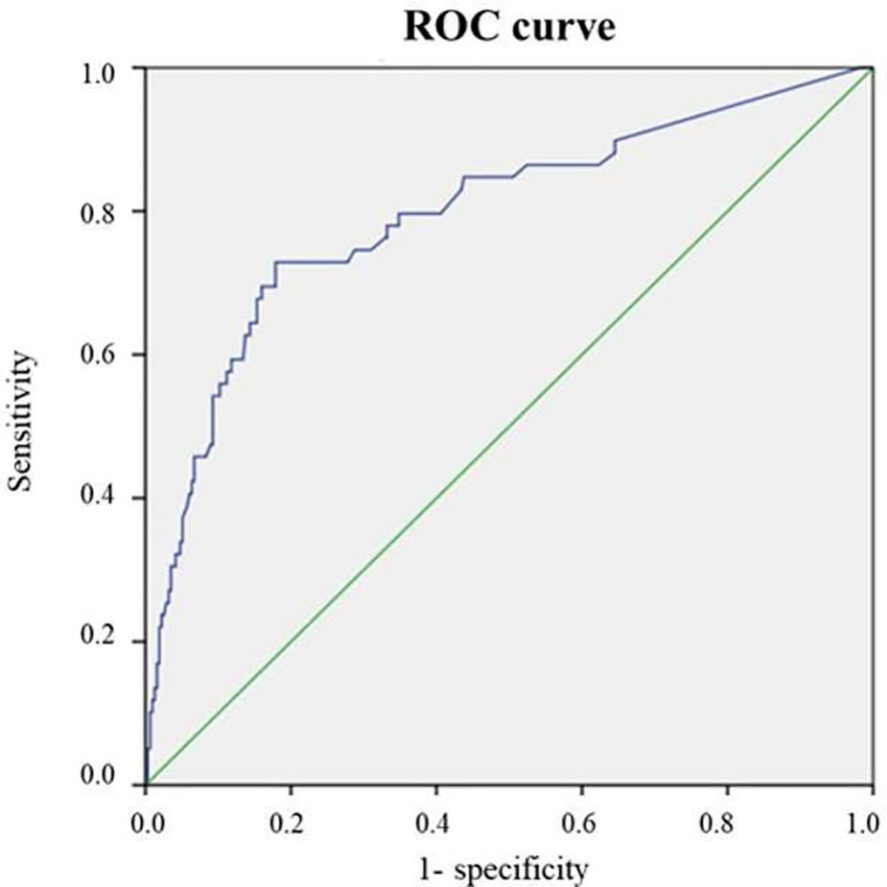
ROC curve of the prognostic model for predicting the risks of radiociodine resistance.

ROC analysis of the discriminative ability of the prognostic model for the development of radioiodine refractoriness, based on multivariate analysis, showed an area under the curve of 0.796 (95% CI 0.726-0.865), p<0.0001. This indicates a good capability of this model to identify patients at risk of developing radioiodine resistance. The Youden’s index cutoff is 0.67, which corresponds to sensitivity and specificity level of 86.4% and 46.3% respectively (**Figure 3**).

Taking into account the influence of gender on the development of radioiodine resistance, we performed a univariate regression analysis in the groups of men and women (**Table 6**). It was found that total thyroidectomy with radical lymph node dissection was associated with radioiodine refractoriness in both men and women, although the association was stronger in women (p < 0.0001 and p = 0.093, respectively). The same situation was typical for both genders with respect to the number of lymph nodes with metastases, the total number of removed lymph nodes, and the total cumulative dose of radiation during radioiodine therapy. The absence of distant metastases had an inverse relationship with the development of radioiodine resistance for both genders (p=0.01; p=0.024, respectively).

**Table 6:** Logistic regression analysis of the risk of developing radioiodine refractoriness by patient gender.

Parameter	Univariate analysis, OR (95% CI)	p	Men	r	Women	r
Complete thyroidectomy with radical lymph node dissection	1.506 (1.246-1.82)	< 0.0001	1,404 (0.945-2.086)	0.093	1.532 (1.231-1.906)	< 0.0001
Total number of LU with metastases	1.16 (1.086-1.238)	< 0.0001	1.123 (1.014-1.244)	0.025	1.165 (1.069-1.269)	< 0.0001
Total number of removed lymph nodes	1.044 (1.016-1.074)	0.002	1.061 (1.001-1.125)	0.045	1,034 (1.0-1.069)	0.048
Absence of distant metastases	0.261 (0.125 -0.543)	< 0.0001	0.132 (0.028-0.621)	0.01	0.357 (0.146-0.871)	0.024
SOD GBk	1.231 (1.152-1.315)	< 0.0001	1.408 (1.160-1.710)	0.001	1.195 (1.113-1.282)	< 0.0001

#### 3.4.1 Resume

The constructed prognostic model demonstrated good discriminatory ability (AUC = 0.796) and can be used to assess the risk of developing radioiodine resistance in patients with differentiated thyroid cancer.

### 3.5 Molecular genetic characteristics of tumors

In the histological subcohort (n = 167), we evaluated mutations that could be associated with the development of radioiodine resistance, comparing their distribution between patients with differentiated thyroid cancer (DTC) in the radioiodine-resistant DTC (RAIR-DTC) group (n = 37) and the radioiodine-sensitive DTC group (n = 130).

No detectable mutations were observed in 33 patients (89.2%) in the RAIR-DTC group, compared with 103 patients (79.2%) in the radioiodine-sensitive DTC group. The difference was not statistically significant (p = 0.169). The BRAF mutation was detected in 33 patients (89.2%) in the RAIR-DTC group and in 105 patients (80.8%) in the radioiodine-sensitive DTC group; again, there were no significant intergroup differences (p = 0.233).

Isolated TERT promoter or NRAS mutations were not identified in either group. Notably, BRAF and TERT promoter mutations (BRAF+pTERT) coexisted exclusively among RAIR-DTC patients, occurring in 12 cases (32.4%). No such cases were observed in the radioiodine-sensitive DTC group (0%). This difference was statistically significant (p < 0.001) (**Table 7**).

**Table 7:** Mutation status (BRAF, рTERT, NRAS) in the RAIR-DTC и RAI-sensitive DTC groups.

Parameters	RAIR-DTC (N = 37)	RAI-sensitivity DTC (N = 130)	p value
No mutation	33.0 (89.2%)	103.0 (79.2%)	0.169^1^
BRAF mutation only	33.0 (89.2%)	105.0 (80.8%)	0.233^1^
pTERT mutation only	0	0	NA
NRAS mutation only	0	0	NA
BRAF+pTERT mutations	12.0 (32.4%)	0.0 (0.0%)	< 0.001^1^

^1^Pearson’s Chi-squared test.

#### 3.5.1 Resume

Across most analyzed mutational categories, no statistically significant differences were observed between the groups. However, the combined presence of BRAF and TERT promoter mutations was significantly associated with radioiodine resistance.

## 4 Discussion

This article presents the results of a case-control study to determine the relationship between the clinical and pathological characteristics of differentiated thyroid carcinoma and the risk of developing resistance to treatment with radioactive iodine I-131. The main study group was represented by 60 patients with radioiodine resistance developed during treatment; the control group included 313 patients classified by age, BMI, treatment principles, and previous thyroid diseases sensitive to radioactive iodine. The strength of our study is the fact that we used a single database for the Republic of Kazakhstan, which determines the use of uniform approaches and algorithms for diagnosis and treatment for all patients.

It should be noted that total thyroidectomy with radical lymph node dissection was performed twice as often in the radioiodine-refractory patient group, indicating more aggressive tumor invasion in this category of patients (p < 0.001). This is further supported by the fact that distant metastases were statistically more frequent in the main study group (p < 0.01).

The results of regression analysis allowed us to establish that the main factors determining the risk of developing radioiodine resistance in patients included in our study were women, total thyroidectomy with radical lymph node dissection, presence of lymph node metastases, cumulative total dose of radioiodine, absence of distant metastases, and the total number of removed lymph nodes. However, in multivariate regression analysis, only total radiation dose, absence of distant metastases, and total number of lymph nodes removed were statistically significant risk factors. These results were confirmed by ROC analysis, which included the risk factors for radioiodine resistance obtained in multi-variate regression analysis. The area under the curve was 0.796 (95% CI 0.726–0.865), p < 0.0001. Considering the influence of gender on the risk of the outcome established in our univariate regression analysis, we performed a stratified adjustment of the studied factors separately for men and women. The results were generally comparable, except for the extent of surgical intervention.

We were particularly interested in comparing our results with similar data from other studies. For example, a case-control study conducted in France in 2024 identified risk factors for the development of radioiodine-refractory thyroid carcinoma. Distant metastases were most common in the lungs, followed by the neck and bones. Based on a multivariate logistic regression model, the following independent risk factors were identified: age at diagnosis ≥ 55, vascular invasion, metastases to the cervical spine, lungs, and bones. The prognostic assessment demonstrated high discriminatory ability of the model with an area under the curve (AUC) of 0.95 (86% sensitivity and 92% specificity) (24).

In another study conducted by Chinese scientists, a scoring system of risk factors for radioiodine resistance was created, which included the following seven independent clinical factors as: age at diagnosis ≥55; the presence of metastases in the neck, lungs and bones during the initial examination; recurrence of metastases in the neck and lungs after treatment (24). These data correlate with our results, except for the age factor, as well as with the results of other authors (25). In the study by Ito Y et al. (2014), independent risk factors for decreased radioactive iodine absorption were patient aged over 60 and male gender (26). Moreover, in the study by Chinese scientists, the risks of radioiodine resistance increased already at the age of patients over 48 years (27).

Patients with radioiodine-resistant forms of DTC receive a significantly higher cumulative dose of radioactive iodine and a greater number of courses of radioactive iodine therapy (28).

A systematic review with meta-analysis performed in 2020 found that tumor extension beyond the thyroid gland and certain histological types of thyroid carcinoma (e.g., high-cell thyroid carcinoma or diffuse sclerosing thyroid carcinoma) increase the risks of developing radioiodine therapy refractory syndrome (29). However, the analysis did not establish a relationship between the development of radioiodine resistance and gender, age, tumor size, or the presence of metastases (30). Nevertheless, the authors associated the obtained results with the heterogeneity of the analyzed studies and small sample sizes in some of them.

In our study, gender was associated with radioiodine resistance only in a univariate model, and the lack of data on hormone receptor expression did not allow for a more detailed assessment of this aspect.

Unlike a number of publications, where age over 45–55 years was considered an unfavorable factor associated with the development of radioiodine resistance in differentiated thyroid cancer, in our cohort, age did not show a statistically significant association with radioiodine resistance in either univariate or multivariate analysis. This is probably due to the characteristics of the sample structure (predominance of middle-aged and elderly patients) and the fact that the influence of age is partially mediated by more strong clinical and pathological predictors, such as the presence of metastases and the parameters of radioiodine therapy included in the regression model.

The size of the malignant neoplasm more than 40 mm, tumor extension beyond the gland, and age of more than 55 were also considered as predictors of thyroid resistance to radioactive iodine (31). Thus, the results of our study are generally consistent with the data obtained by other authors under similar conditions (32).

In the histological subcohort (n = 167), we evaluated mutations potentially associated with the development of radioiodine resistance, including BRAF V600E, NRAS (codon 61), and TERT promoter mutations (C228T and C250T). Then we compared their distribution between patients with DTC classified as RAIR-DTC (n = 37) and RAI-sensitive DTC (n = 130).

The observed distribution of mutations indicates that the presence of a single BRAF mutation is not sufficient to identify radioiodine resistance in the studied cohort. By contrast, a defining feature of the RAIR group was the occurrence of combined BRAF and TERT promoter mutations. This is consistent with the idea that driver events can work together to enhance tumor aggressiveness and cause a loss of radioiodine-avid function. This finding is clinically important, as identifying coexisting mutations could have prognostic value and facilitate the earlier identification of patients at increased risk of developing the RAIR phenotype. This would support closer surveillance and the earlier consideration of alternative therapeutic strategies.

## 5 Limitations

The main limitation of our study was the fact that in the Republic of Kazakhstan, treatment of DTC began relatively recently (within ten years). Therefore, the study included all patients who underwent treatment during this period of time. The most appropriate design for studying the risk factors for the development of radioiodine resistance could be a cohort study, but in current conditions, we did not accumulate sufficient material to conduct such a study; therefore, a case-control study was conducted.

Molecular analysis was performed on a histological subcohort of patients for whom archival material was available. An in-depth analysis of this subcohort’s molecular genetic profile is currently underway. The final results will be presented upon completion of the comprehensive analysis.

## 6 Conclusions

The development of resistance to radioactive iodine therapy in differentiated thyroid cancer (DTC) is one of the most pressing challenges in modern oncology. This is due to the significant social impact of the disease and the substantial resources required for therapy, diagnosis and long-term monitoring of treatment effectiveness. Our study indicates that the main risk factors for developing resistance to radioactive iodine therapy are female sex; total thyroidectomy with compartment-oriented neck dissection; the presence of lymph node metastases; the cumulative administered radioiodine dose; the absence of distant metastases; and the total number of removed lymph nodes. Additionally, within the histological subcohort (n = 167), the presence of both BRAF and TERT promoter mutations was identified as a molecular factor associated with radioiodine resistance. Integrating clinical and molecular data could enable more accurate risk stratification for radioiodine resistance and inform future research directions.

## Data Availability

The raw data supporting the conclusions of this article will be made available by the authors, without undue reservation.

## References

[1] Kitahara CM Sosa JA . The changing incidence of thyroid cancer. Nat Rev Endocrinol. (2016) 12:646–53. doi: 10.1038/nrendo.2016.110, PMID: 27418023 PMC10311569

[2] Raue F Frank-Raue K . Thyroid cancer: risk-stratified management and individualized therapy. Clin Cancer Res. (2016) 22:5012–21. doi: 10.1158/1078-0432.CCR-16-0484, PMID: 27742787

[3] American Cancer Society . (2021). Available online at: https://www.cancer.org/ (Accessed July 2, 2025).

[4] Hu S Wu X Jiang H . Trends and projections of the global burden of thyroid cancer from 1990 to 2030. J Glob Health. (2024) 14:4084. doi: 10.7189/jogh.14.04084, PMID: 38751316 PMC11109522

[5] Siegel RL Miller KD Jemal A . Cancer statistics, 2016. CA Cancer J Clin. (2016) 66:7–30. doi: 10.3322/caac.21332, PMID: 26742998

[6] Fagin JA Wells SA Jr . Biological and clinical perspectives on thyroid cancer. N Engl J Med. (2016) 375:1054–67. doi: 10.1056/NEJMra1501993, PMID: 27626519 PMC5512163

[7] Wang LY Palmer FL Nixon IJ Thomas D Patel SG Shaha AR . Multi-organ distant metastases confer worse disease- specific survival in differentiated thyroid cancer. Thyroid. (2014) 24:1594–9. doi: 10.1089/thy.2014.0173, PMID: 25162180

[8] Haq M Harmer C . Differentiated thyroid carcinoma with distant metastases at presentation: prognostic factors and outcome. Clin Endocrinol (Oxf). (2005) 63:87–93. doi: 10.1111/j.1365-2265.2005.02304.x, PMID: 15963067

[9] Ibrahim EY Busaidy NL . Treatment and surveillance of advanced, metastatic iodine-resistant differentiated thyroid cancer. Curr Opin Oncol. (2017) 29:151–8. doi: 10.1097/CCO.0000000000000349, PMID: 28141684

[10] Capdevila J Argiles G Rodriguez-Frexinos V Nuñez I Tabernero J . New approaches in the management of radioiodine-resistance thyroid cancer: the molecular targeted therapy era. Discov Med. (2010) 9:153–62. 20193642

[11] Aashiq M Silverman DA Na’ara S Takahashi H Amit M . Radioiodine-resistance thyroid cancer: molecular basis of redifferentiation therapies, management, and novel therapies. Cancers (Basel). (2019) 11:1382. doi: 10.3390/cancers11091382, PMID: 31533238 PMC6770909

[12] Coelho SM Vaisman F Buescu A Mello RC Carvalho DP Vaisman M . Follow-up of patients treated with retinoic acid for the control of radioiodine non-responsive advanced thyroid carcinoma. Braz J Med Biol Res. (2011) 44:73–7. doi: 10.1590/s0100-879x2010007500120, PMID: 21085896

[13] Antonelli A Ferri C Ferrari SM Sebastiani M Colaci M Ruffilli I . New targeted molecular therapies for dedifferentiated thyroid cancer. J Oncol. (2010) 921682. doi: 10.1155/2010/921682, PMID: 20628483 PMC2902220

[14] Durante C Haddy N Baudin E Leboulleux S Hartl D Travagli JP . Long-term outcome of 444 patients with distant metastases from papillary and follicular thyroid carcinoma: benefits and limits of radioiodine therapy. J Clin Endocrinol Metab. (2006) 91:2892–9. doi: 10.1210/jc.2005-2838, PMID: 16684830

[15] Wassermann J Bernier MO Spano JP Lepoutre-Lussey C Buffet C Simon JM . Outcomes and prognostic factors in radioiodine refractory differentiated thyroid carcinomas. Oncologist. (2016) 21:50–8. doi: 10.1634/theoncologist.2015-0107, PMID: 26675742 PMC4709201

[16] Schlumberger M Tahara M Wirth LJ Robinson B Brose MS Elisei R . Lenvatinib versus placebo in radioiodine-refractory thyroid cancer. N Engl J Med. (2015) 372:621–30. doi: 10.1056/NEJMoa1406470, PMID: 25671254

[17] Shen H Zhu R Liu Y Hong Y Ge J Xuan J . Radioiodine-refractory differentiated thyroid cancer: Molecular mechanisms and therapeutic strategies for radioiodine resistance. Drug Resist Updat. (2024) 72:101013. doi: 10.1016/j.drup.2023.101013, PMID: 38041877

[18] Scheffel RS Zanella AB Dora JM Maia AL . Timing of radioactive iodine administration does not influence outcomes in patients with differentiated thyroid carcinoma. Thyroid. (2016) 26:1623–9. doi: 10.1089/thy.2016.0038, PMID: 27549175

[19] Haugen BR Alexander EK Bible KC Doherty GM Mandel SJ Nikiforov YE . 2015 American thyroid association management guidelines for adult patients with thyroid nodules and differentiated thyroid cancer: the american thyroid association guidelines task force on thyroid nodules and differentiated thyroid cancer. Thyroid. (2016) 26:1–133. doi: 10.1089/thy.2015.0020, PMID: 26462967 PMC4739132

[20] Boudina M . Radioiodine refractory differentiated thyroid cancer. Hell J Nucl Med. (2023) 26 Suppl:65–8. 37658568

[21] Ringel MD Sosa JA Baloch Z Bischoff L Bloom G Brent GA . 2025 American thyroid association management guidelines for adult patients with differentiated thyroid cancer. Thyroid. (2025) 35:841–985. doi: 10.1177/10507256251363120, PMID: 40844370 PMC13090833

[22] Basolo F Macerola E Poma AM Torregrossa L . The 5th edition of WHO classification of tumors of endocrine organs: changes in the diagnosis of follicular-derived thyroid carcinoma. Endocrine. (2023) 80:470–6. doi: 10.1007/s12020-023-03336-4, PMID: 36964880 PMC10199828

[23] Haddad RI Bischoff L Ball D Bernet V Blomain E Busaidy NL . Thyroid carcinoma, version 2.2022, NCCN clinical practice guidelines in oncology. J Natl Compr Canc Netw. (2022) 20:925–51. doi: 10.6004/jnccn.2022.0040, PMID: 35948029

[24] Li G Lei J Song L Jiang K Wei T Li Z . Radioiodine refractoriness score: A multivariable prediction model for postoperative radioiodine-refractory differentiated thyroid carcinomas. Cancer Med. (2018) 7:5448–56. doi: 10.1002/cam4.1794, PMID: 30264548 PMC6246937

[25] Kersting D Seifert R Kessler L Herrmann K Theurer S Brandenburg T . Predictive factors for RAI-refractory disease and short overall survival in PDTC. Cancers (Basel). (2021) 13:1728. doi: 10.3390/cancers13071728, PMID: 33917322 PMC8038667

[26] Schubert L Mbekwe-Yepnang AM Wassermann J Braik-Djellas Y Jaffrelot L Pani F . Clinico-pathological factors associated with radioiodine refractory differentiated thyroid carcinoma status. J Endocrinol Invest. (2024) 47:1573–81. doi: 10.1007/s40618-024-02352-z, PMID: 38578580 PMC11143047

[27] Ito Y Miyauchi A Ito M Yabuta T Masuoka H Higashiyama T . Prognosis and prognostic factors of differentiated thyroid carcinoma after the appearance of metastasis refractory to radioactive iodine therapy. Endocr J. (2014) 61:821–4. doi: 10.1507/endocrj.ej14-0181, PMID: 24871888

[28] Liu Y Wang Y Zhang W . Scoring system and a simple nomogram for predicting radioiodine refractory differentiated thyroid cancer: a retrospective study. EJNMMI Res. (2022) 12:45. doi: 10.1186/s13550-022-00917-8, PMID: 35904608 PMC9338217

[29] Shobab L Gomes-Lima C Zeymo A Feldman R Jonklaas J Wartofsky L . Clinical, pathological, and molecular profiling of radioactive iodine refractory differentiated thyroid cancer. Thyroid. (2019) 29:1262–8. doi: 10.1089/thy.2019.0075, PMID: 31319763

[30] Luo Y Jiang H Xu W Wang X Ma B Liao T . Clinical, pathological, and molecular characteristics correlating to the occurrence of radioiodine refractory differentiated thyroid carcinoma: A systematic review and meta-analysis. Front Oncol. (2020) 10:549882. doi: 10.3389/fonc.2020.549882, PMID: 33117686 PMC7561400

[31] Lorusso L Minaldi E Esposito G Piaggi P Bottici V Brogioni S . Radio-iodine refractory thyroid cancer patients: a tailored follow-up based on clinicopathological features. J Endocrinol Invest. (2023) 46:2165–73. doi: 10.1007/s40618-023-02076-6, PMID: 37084131 PMC10514097

[32] Chereau N Oyekunle TO Zambeli-Ljepović A Kazaure HS Roman SA Menegaux F . Predicting recurrence of papillary thyroid cancer using the eighth edition of the AJCC/UICC staging system. Br J Surg. (2019) 106:889–97. doi: 10.1002/bjs.11145, PMID: 31012500 PMC6825520

